# In silico-guided engineering of *Pseudomonas putida* towards growth under micro-oxic conditions

**DOI:** 10.1186/s12934-019-1227-5

**Published:** 2019-10-22

**Authors:** Linde F. C. Kampers, Ruben G. A. van Heck, Stefano Donati, Edoardo Saccenti, Rita J. M. Volkers, Peter J. Schaap, Maria Suarez-Diez, Pablo I. Nikel, Vitor A. P. Martins dos Santos

**Affiliations:** 1 Systems and Synthetic Biology, Wageningen University and Research Centre, Stippeneng 4, 6708 WE Wageningen, The Netherlands; 2 Max Planck Institute for Terrestrial Microbiology, Karl-von-Frisch-Strasse 16, 35043 Marburg, Germany; 3 The Novo Nordisk Foundation Center for Biosustainability, Kgs Lyngby, Denmark; 4 LifeGlimmer GmbH, Berlin, Germany

**Keywords:** Synthetic biology, Constraint-based metabolic modelling, Comparative genomics, Domainome analysis, Anaerobiosis, Microbial physiology

## Abstract

**Background:**

*Pseudomonas putida* is a metabolically versatile, genetically accessible, and stress-robust species with outstanding potential to be used as a workhorse for industrial applications. While industry recognises the importance of robustness under micro-oxic conditions for a stable production process, the obligate aerobic nature of *P. putida*, attributed to its inability to produce sufficient ATP and maintain its redox balance without molecular oxygen, severely limits its use for biotechnology applications.

**Results:**

Here, a combination of genome-scale metabolic modelling and comparative genomics is used to pinpoint essential $\text {O}_{2}$-dependent processes. These explain the inability of the strain to grow under anoxic conditions: a deficient ATP generation and an inability to synthesize essential metabolites. Based on this, several *P. putida* recombinant strains were constructed harbouring acetate kinase from* Escherichia coli* for ATP production, and a class I dihydroorotate dehydrogenase and a class III anaerobic ribonucleotide triphosphate reductase from *Lactobacillus lactis* for the synthesis of essential metabolites. Initial computational designs were fine-tuned by means of adaptive laboratory evolution.

**Conclusions:**

We demonstrated the value of combining in silico approaches, experimental validation and adaptive laboratory evolution for microbial design by making the strictly aerobic *Pseudomonas putida* able to grow under micro-oxic conditions.

## Introduction

The *Pseudomonas* genus is known for its metabolic versatility, high supply of reducing power, and tolerance to toxins and solvents [1–3]. In particular, *Pseudomonas putida* KT2440 is HV1 certified (safe to use in an ML1 or P1 environment), genetically accessible [4–9], and has been successfully engineered to produce various compounds of industrial interest [10, 11]. Therefore, *P. putida* KT2440 is a recognized synthetic biology- and industrial workhorse [4, 10–13]. Genome-scale, constraint-based models of metabolism (GSMs) have been developed to analyse its bio-degradative and biotechnological capacities [4–9]. GSMs provide a comprehensive list of all genome-encoded reactions of an organism and can be used to make predictions of growth in different media and/or environmental conditions [14].

Several species in the *Pseudomonas* genus are facultative anaerobes (e.g *P. aeruginosa, P. fluorescens, P. denitrificans*), suggesting that the strictly aerobic *P. putida* could be designed towards a micro-aerobic or even facultative anaerobic lifestyle with a limited number of genetic modifications. The latter has been experimentally attempted several times by engineering either anaerobic fermentation or anaerobic respiration.

The first attempt to create a *P. putida* strain capable of anaerobic fermentation was by Sohn et al. [6]. They created a GSM of *P. putida* KT2440 from which they concluded that *P. putida* KT2440 cannot grow anaerobically due to insufficient ATP generation. Anaerobic ATP generation was then enhanced through the expression of acetate kinase via a plasmid, which resulted in 10 times more surviving *P. putida* KT2440 cells in minimal medium with glucose as only carbon source after 8 days of exposure to anoxic conditions.

In a later in vivo study, Nikel et al. [15] reasoned that in the absence of aerobic respiration there is not only a lack of ATP generation, but also an accumulation of NADH that cannot be re-oxidized to NAD^+^ via the electron transfer chain. Therefore they expressed acetate kinase, pyruvate decarboxylase and alcohol dehydrogenase II to facilitate energy generation and redox rebalancing, respectively. This approach also yielded approximately 10 times more surviving *P. putida* KT2440 cells compared to the control strain after 7 days of exposure to anoxic conditions, with a viability rate of $58\pm 4\%$ when both these enzymes were expressed versus $37\pm 3\%$ for sole expression of acetate kinase [6, 15].

In a different approach Steen et al. [16] introduced the nitrate or nitrite respiration machinery in *P. putida* KT2440. Nitrate and nitrite respiration are common anaerobic alternatives to $\text {O}_2$ respiration in other *Pseudomonas* species, but are completely absent in *P. putida* strains. Therefore, Steen et al. separately expressed the *nar* or *nir-nor* operons from *P. aeruginosa* ATCC 17933 in *P. putida* KT2440 to enable nitrate or nitrite respiration respectively. *P. putida* KT2440 expressing the *nar *operon yielded a 50-fold higher number of colony forming units (CFUs) compared to the control strain when incubated for 5 days under anoxic conditions in the presence of nitrate, and *P. putida* KT2440 expressing the *nir*-*nor* operon displayed an up to 80-fold higher number of CFUs in the presence of nitrite.

Another approach to attempt anaerobic respiration in *P. putida* was the use of phenazines to transfer electrons from the cell to an electrode, to re-establish functioning of the electron transfer chain under oxygen limiting conditions. Schmitz et al. [17] expressed seven core phenazine biosynthesis genes *phzA-G* and the two specific genes *phzM* and *phzS* required for pyocyanin synthesis from *P. aeruginosa* PAO1 into *P. putida* KT2440. They observed that phenazines facilitate electron discharge to the electrode, although the wild-type* P. putida* KT2440 unexpectedly also showed a limited ability to interact with the electrode. The production of phenazines resulted in sustained metabolism under oxygen-limited conditions for up to 2 weeks, with doubled biomass production in the presence of a poised electrode when compared to wild-type *P. putida* KT2440.

Similarly, Lai et al. [18] showed that adding the redox mediators thionine chloride, tris(2,2′-bipyridine)cobalt(II) diperchlorate, or potassium hexacyano-ferrate(III) to the culture medium could enable wild-type *P. putida* F1 to discharge electrons to an electrode. This resulted in survival of *P. putida* F1 under anoxic conditions over 300 h with a higher adenylate energy charge, indicating the cells could generate energy using the anode as terminal electron acceptor.

The attempts for anaerobic fermentation or anaerobic respiration yielded similar levels of survival under anoxic conditions. None of the studies reported growth of the strains under anoxic conditions [6, 15–18]. Overall, anaerobic fermentation would be preferred over anaerobic respiration since this eliminates the need of supplementing the electron acceptor (oxygen or nitrate/nitrite) homogeneously through the bioreactor, and fermentation often results in products of industrial value such as lactate. Recombinant strain behaviour through fermentation under micro-oxic conditions was not explored in any of the aforementioned studies.

In this work, we explore the roles oxygen plays in *P. putida* KT2440 by focusing on growth under anoxic in silico and micro-oxic conditions in silico and in vivo. In our pursuit of these goals we took advantage of the available knowledge on *P. putida* KT2440 metabolism, as well as of the wealth of genomic data on *P. putida* KT2440 and other *Pseudomonas* species [14]. Specifically, we used a GSM to probe *P. putida* KT2440 metabolism, while comparative genomics was used to pinpoint the distinct genetic features of *Pseudomonas* species capable of growing under anoxic conditions through anaerobic fermentation. These in silico approaches identified several limitations of *P. putida* KT2440 to grow under micro-oxic or anoxic conditions. These limitations were systematically addressed in recombinant *P. putida* KT2440 strains, which were then exposed to micro-oxic conditions through an oxygen gradient set-up to evaluate their capabilities.

## Materials and methods

### Genome-scale, constraint-based metabolic models

In this study we used the *P. putida* KT2440 genome-scale metabolic models iJN746 [5] and iJP962 [7].

### GSM simulations

iJN746 and iJP962 were analysed using Flux Balance Analysis (FBA) in the CobraPy toolbox [14, 19]. Growth predictions were performed by setting biomass production as maximization objective in FBA. The minimal glucose medium conditions were set by allowing ’unlimited’ uptake (up to 1000 mmol/(gdw h), gdw: grams dry weight) of copper, cobalt, iron, protons, water, sodium, nickel, ammonia, phosphate, sulphate, and nitrate. In addition, the glucose uptake rate was set to be maximally 6.14 mmol/(gdw h), based on experimentally measured uptake rates [20]. The in silico medium composition closely resembles the composition of DeBont minimal medium which was used for the in vivo experiments.

Rich medium conditions were set by allowing unlimited uptake (up to 1000 mmol/(gdw h)) of all compounds (except oxygen) for which exchange reactions are present in the GSM. Under oxic conditions, the $\text {O}_{2}$ uptake rate was set to maximally 18.5 mmol/(gdw h). Reactions for previous designs or to complement newly identified reactions were added using the CobraPy ‘addReaction’ function.

### Genome annotation

We selected six genomes of facultative anaerobic strains from the *Pseudomonas* genus (*P. aeruginosa* PAO1 and M18, *P. stutzeri* DSM10701 and A1501, *P. denitrificans* ATCC13867 and *P. fluorescens* F113) and six genomes of obligate aerobic *P. putida* strains (KT2440, F1, S16, W619, GB1, and BIRD1). All genomes were obtained from the EnsemblBacteria repository in March 2015 [21]. Genomes were annotated in SAPP [22, 23] using Prodigal for gene prediction (version 2.6) [24], 2010 and with InterProScan version 5.4-47.0 [25] for functional annotation, with the selected applications: TIGRFAM, ProDom, SMART, PROSITE Pattern, PfamA, PRINTS, SUPERFAMILY, Coils, Gene3d.

### Bacterial strains and cultivation conditions

Bacterial strains used in this study can be found in Additional file 1: Table S1. *E. coli* $\text {CC118}\lambda \text {pir}$ was used for cloning procedures and plasmid maintenance, and was routinely cultivated at 37 °C under oxic conditions in LB medium (containing 10 g/l tryptone, 10 g/l NaCl and 5 g/l yeast extract), optionally containing antibiotics for selection (50 μg/ml kanamycin as indicated). For solid medium, 15 g/l agar was added to the medium. *P. putida* KT2440 was routinely cultivated under oxic conditions at $30^\circ \text {C}$ in LB medium. Growth and fluorescence experiments were performed in De Bont minimal medium (DBG) [26] (3.88 g/l $\text {K}_{2}\text {HPO}_{4}$, 1.63 g/l $\text {NaH}_{2}\text {PO}_{4}\cdot \,\text {2H}_{2}\text {O}$, 2.00 g/l $(\text {NH}_{4})_{2}\text {SO}_{4}$, 0.1 g/l $\text {MgCl}_{2}\cdot 6\text {H}_{2}\text {O}$, 10 mg/l EDTA, 2 mg/l $\text {ZnSO}_{4}\cdot \,7\text {H}_{2}\text {O}$, 1 mg/l $\text {CaCl}_{2}$ $\cdot \,2\text {H}_{2}\text {O}$, 5 mg/l $\text {FeSO}_{4}\cdot \, 7\text {H}_{2}\text {O}$, 0.2 mg/l $\text {Na}_{2}\text {MoO}_{4}\cdot 2\text {H}_{2}\text {O}$, 0.2 mg/l $\text {CuSO}_{4}\cdot 5\text {H}_{2}\text {O}$, 0.4 mg/l $\text {CoCl}_{2}\cdot 6\text {H}_{2}\text {O}$, 1 mg/l $\text {MnCl}_{2}\cdot 2\text {H}_{2}\text {O}$), with 4.5 g/l glucose as the sole carbon source. The medium was supplemented with 50 μg/ml kanamycin as indicated. Pre-cultures were prepared by picking and transferring a single colony from solid LB medium with the appropriate antibiotic selection into culture flasks for oxic overnight cultivation at 200 rpm at indicated cultivation temperatures.

### Construction of plasmids

Plasmids used in this study are described in Additional file 1: Table S1. DNA segments were amplified from the indicated template by colony-PCR using Phire Hot Start II DNA Polymerase (Thermo Fisher Scientific, Waltham, MA, USA) according to the manufacturers’ protocol (primers are listed in Additional file 2: Table S2). Restriction enzymes were obtained from NEB (New England $\text {BioLabs}\circledR \text {inc}$, Ipswich, MA USA.). l-aspartate oxidase was isolated from both *E. coli* BW25113 or *P. putida* KT2440. Both genes were designed with NdeI and BamHI restriction sites on the 5′- and 3′-ends, respectively. DNA fragments were purified from agarose gel using the KG Gel Purification Kit (Machery-Nagel GmbH and Co. Düren, Germany) and ligated into the pSEVA638 backbone (Standardized SEVA plasmid system [27, 28]) using T4 DNA Ligase (Roche Applied Science, Indianapolis, IN USA). Acetate kinase (*ackA*) was isolated from *E. coli*, the two-subunit gene cluster class I dihydroorotate hydrogenase (*pyrK-pyrD B*) and the two-subunit gene cluster class III ribonucleotide triphosphate reductase (*nrdD-nrdG*) were ordered using the gene from *L. lactis* as a template but codon-optimized for *P. putida* KT2440. The genes were designed with PacI and SpeI restriction sites on the 5′- and 3′-ends, respectively. Similarly purified DNA segments were ligated into the pSEVA2213 backbone (Standardized SEVA plasmid system [27, 28]). Each construct additional carries the fluorescent protein EcFbFP [29] which does not depend on oxygen to check if the genes are transcribed and in roughly what amount the proteins are present. The empty plasmid control only carries *EcFbFP*.

### Micro-oxic experiments

Oxygen gradients are based on Bailey and Scott’s Diagnostic Microbiology [30]. Oxygen gradients were set up in sterile 10 ml glass test tubes capped with loosely fitting caps and filled halfway with LB medium. After autoclaving, the medium was supplemented with sterile components: antibiotics as indicated, 5 g/l l-cysteine and 0.5 g/l sodium thioglycollate to remove $\text {O}_2$ from the medium, 1 mg/l resazurin to indicate $\text {O}_{2}$ presence and 4 g/l agarose to stabilize the gradients. After heating the medium to just below boiling point for 10 min to expel $\text {O}_{2}$, the tubes were cooled to room temperature. 24 h After preparation, the tubes were inoculated drop-wise with 10 μl of a pre-culture.

Strain performance in oxygen gradients was tested while oxygen gradient test tubes were slanted at a $45^\circ$ angle or upright. Multiple agar or agarose concentrations and variants were tested (Additional file 3: Table S4, Additional file 4: Table S5). Micro-oxic conditions within the gradient were verified with a micro-electrode (similar set-up as described in [31, 32]). The resazurin colour change from pink to colourless coincides with a dissolved oxygen concentration around the micro-electrode detection limit of 0.01 mg/l (Additional file 5: Figure S1). Growth of the strains through the oxygen gradient was monitored via a Mirazoom MZ-902 time-lapse camera (OOWL tech, Kowloon, Hong Kong) set-up in a well-lit non-shaking 30 °C incubator. Time-lapse movies were analysed using Fiji 1.51p (ImageJ 1.51p, Rasband, National institutes of Health, Bethesda, MA USA) (Additional file 6: Analysis S1). Growth in the oxygen gradients started from the surface of the medium (high in oxygen) downwards (gradually lower in oxygen), and was measured in mm. Sampling of bacteria was done by selecting the bacteria growing at the lowest oxygen concentration with a 3 ml syringe (Thermo Fisher Scientific, Waltham, MA USA) and 1.5″ needle (BD Microlance, Switzerland).

### Anoxic cultivation

Anoxic cultivation of *E. coli* JW2558 was performed in 50 ml glass 20 mm aluminium crimp cap vials with rubber stoppers (Glasgerätebau Ochs laborfachhandel e.K.) in 30 ml DBG medium supplemented with 0.75 g/l l-cysteine, 1 mg/l resazurin and with 50 μg/ml gentamycin as selection marker. Before inoculation, the vials were gas exchanged with $\text {CO}_2/\text {N}_2$. Inoculation was done with aerobically precultured bacterial sample, with a starting OD of ca. 0.03. Samples were taken using $\text {CO}_2$ flushed 1.5″ needles (BD Microlance) and 3–5 ml syringes (ThermoFisher) avoiding $\text {O}_2$ exposure. Anoxic conditions were ensured as in samples taken the resazurin turned from colourless to bright pink within seconds. Growth rates were determined by $\text {OD}_{600}$ measurements. Medium only served as a blank control.

### Statistical analysis

All reported experiments were independently repeated three times, testing biological triplicates. Figures represent the mean values of corresponding biological triplicates and the standard deviation. The level of significance of the differences when comparing results was evaluated by means of analysis of variance (ANOVA), with $\alpha =0.05$.

## Results

### Model evaluation confirms the strict aerobic nature of *P. putida *KT2440

Previous designs of anaerobically surviving *P. putida* strains were conceptually based on insufficient anaerobic ATP generation and redox balancing [6, 15–18]. Implementation of acetate kinase resulted in prolonged survival times under anoxic conditions [6, 15, 16]. Therefore, we first re-evaluated these designs in the context of the *P. putida* KT2440 models iJP962 [7] and iJN746 [5]. GSMs iJP962 and iJN746 describe the known metabolism of *P. putida* KT2440 as well as its requirements for survival and growth. When analysed with Flux Balance Analysis (FBA) [14], these GSMs can predict whether or not *P. putida* grows under various conditions [5, 7]. The analysis of the two GSMs led to similar results and hereafter we will specifically report those obtained with iJP962.

iJP962 was first confirmed to correctly predict the obligate aerobic nature of wild-type *P. putida* KT2440. It predicted the maximal achievable growth rate under anoxic conditions in both rich and minimal medium to be zero, which is in line with the obligate aerobic nature of *P. putida*. iJP962 Was then used to contextualize the anaerobic *P. putida* designs referred to above. The GSM was expanded with reactions corresponding to the expressed heterologous genes from previous experimental designs [6, 15–17]. These expanded GSMs still predicted that anaerobic growth was not possible in neither minimal nor rich medium. These predictions are consistent with the experimental observations that none of the previous designs enabled *P. putida *KT2440 to grow anaerobically. In addition, these predictions imply that iJP962 captures previously undescribed limitations to anaerobic growth in *P. putida *KT2440.

### Limited energy supply and inability to generate biomass inhibit *P. putida* to survive under anoxic conditions

**Fig. 1:**
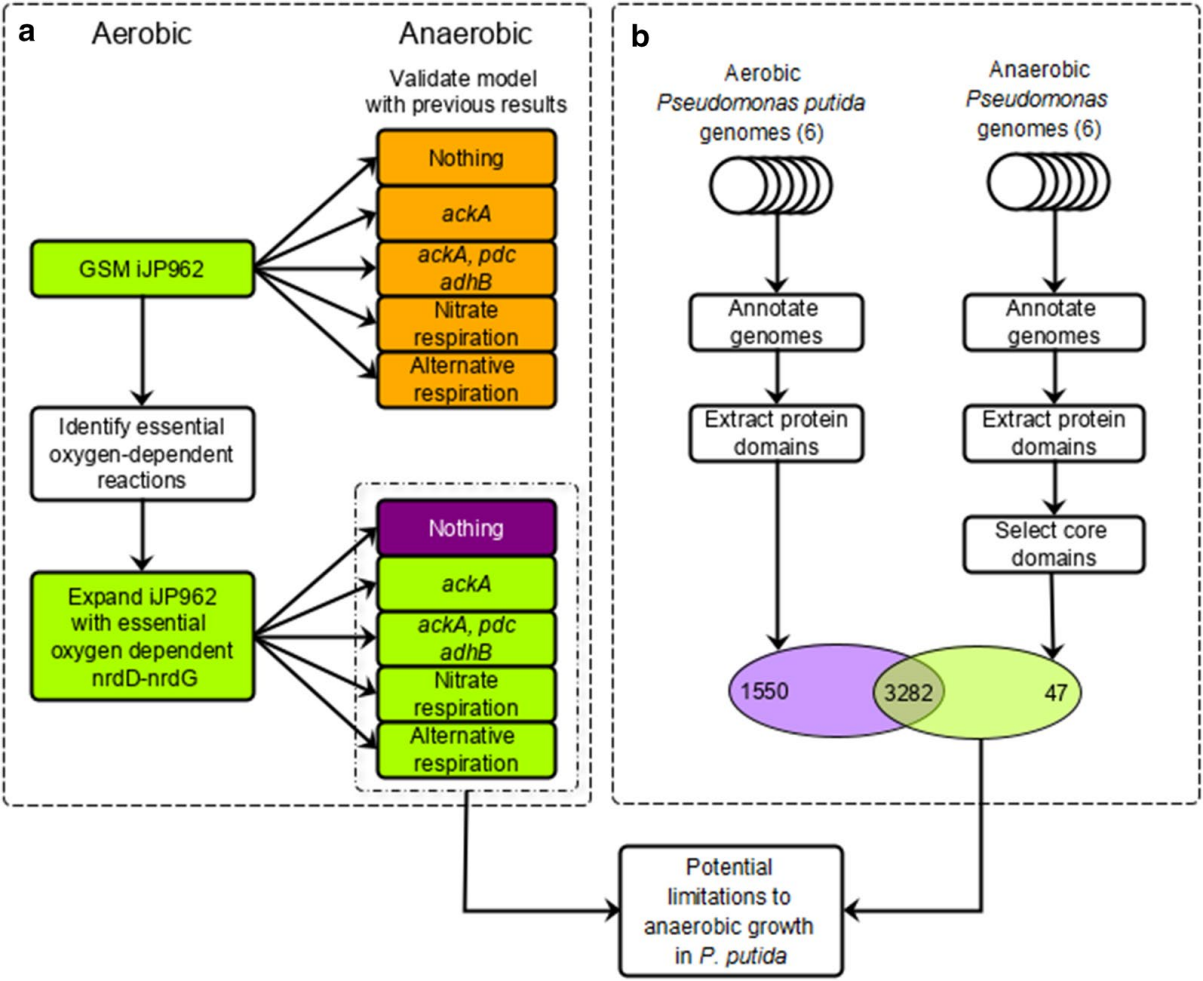
Overview of in silico approaches to identify limitations to anaerobic growth in *P. putida*. **a** Summary of genome-scale GSM predictions using iJP962 [7] given an aerobic environment (left) and expansions with indicated reaction sets given an anaerobic environment (right). The colours indicate no growth (orange), poor growth (purple) and growth (green). **b** Comparative genomics workflow. Genomes of the* P. putida* group and the anaerobic *Pseudomonas* group were systematically annotated using SAPP [22, 23], the protein domains were extracted, and the domains common to all anaerobic *Pseudomonas* species (the core domains) were selected. Predictions from both methods were combined to obtain a final design

**Fig. 2:**
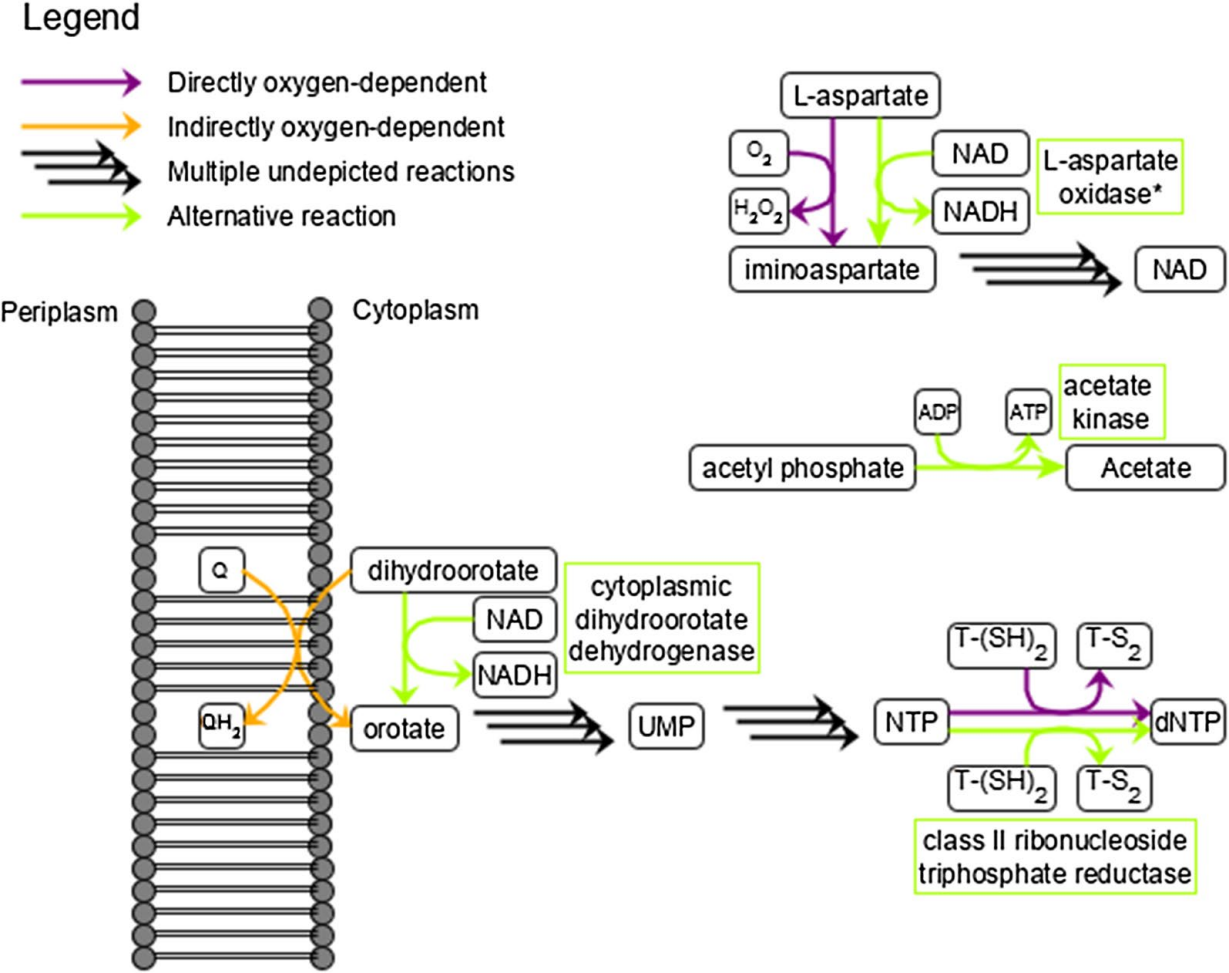
Final design of *P. putida* KT2440 capable of growth under anoxic conditions through anaerobic fermentation. *QH2* quinol, *Q* quinone, *T-(SH)2* reduced thioredoxin, T-S2 oxidized thioredoxin, *NTP* nucleoside triphosphate, *dNTP* deoxynucleoside triphosphate, *UMP* uridine monophosphate

The model allows identification of reactions which indirectly use oxygen, thereby resulting in limited survival under micro-oxic conditions. To identify additional limitations, we performed two independent in silico analyses: GSM simulations and domainome, comparative genomic analysis. The GSM simulation approach focused on identifying essential $\text {O}_2$-dependent metabolic reactions in iJP962, whereas the comparative genomics approach focused on pinpointing the genetic differences between obligate aerobic *P. putida* strains and other facultative anaerobic *Pseudomonas* species (Fig. 1). To identify reactions in the GSM that involve $\text {O}_2$ and are essential for growth, we first set the growth medium to a minimal glucose medium under oxic conditions. Then, we iteratively deleted each reaction that involves $\text {O}_2$ one at a time and predicted whether or not growth was possible. Growth was no longer possible upon the deletion of either (i) protoporphyrinogen oxidase, (ii) l-aspartate oxidase, or (iii) dihydroorotate dehydrogenase. These enzymes are required for the biosynthesis of heme, NAD^+^/NADP^+^, and pyrimidines, respectively (Fig. 2).

Next, we evaluated whether the lack of anaerobic alternatives to these three reactions is the only limitation to in silico anaerobic growth. We expanded iJP962 with anaerobic alternatives for l-aspartate oxidase, dihydroorotate dehydrogenase and protoporphyrinogen oxidase and again simulated growth. iJP962 now predicted anaerobic growth of the modified *P. putida* on glucose minimal medium, suggesting that the lack of anaerobic alternatives to the aforementioned three reactions is the only limitation to anaerobic growth.

The predicted anaerobic growth rate with the three aforementioned metabolic alternatives was, however, very low (0.007 h^−1^) compared to the aerobic growth rate using the unamended model (0.450 h^−1^). Although low, this does suggest that according to the GSM the modified *P. putida* is capable of producing all biomass constituents necessary for growth. The comparatively low growth rate suggests a lack of resources or an inefficient conversion thereof.

The limiting factor was identified to be ATP formation. Under anoxic conditions, most ATP required for biomass formation is in silico generated by *oxygen-dependent* cytochrome reactions. Therefore, we further expanded iJP962 with reactions corresponding to the previous anaerobic *P. putida* designs that dealt with anaerobic ATP generation [6, 15–17]. The addition of acetate kinase to the model doubled the predicted growth rate to 0.014 $\text {h}^{-1}$, while addition of the nitrate respiration machinery increased the predicted growth rate to 0.171 $\text {h}^{-1}$. Based on the modelling results, we hypothesized that the anaerobic ATP generation is insufficient.

Although the GSM-based approach successfully identified several limitations to anaerobic growth in *P. putida*, this identification is restricted to metabolism as described in iJP962. Other cellular processes that may rely on $\text {O}_2$ are not described. Therefore, we also used comparative genomics to perform an analysis of protein domain content and pinpoint genetic differences between select groups of obligate aerobic *P. putida* strains and facultative anaerobic *Pseudomonads*. The *P. putida* group consisted of the following strains: KT2440, F1, S16, W619, GB1, and BIRD1. The facultative anaerobic *Pseudomonas* group consisted of *P. aeruginosa* PAO1 and M18, *P. stutzeri* DSM10701 and A1501, *P. denitrificans* ATCC13867 and *P. fluorescens* F113.

All genomes were de novo annotated to avoid artifacts from differences in the annotation procedures. Annotated protein domains were extracted from each genome to compare the presence of functional protein domains. The domains from the anaerobic *Pseudomonas* group were then further filtered to select only those domains that are shared by all members of the group: core domains. These core domains were compared to the domains present in the *P. putida* strains to identify domains common to all selected anaerobic *Pseudomonas* species and absent from all selected *P. putida* strains. This resulted in a shortlist of 47 anaerobic-only protein domains (Fig. 1, Additional file 7: Table S3).

These domains thus identify the genetic makeup of the anaerobic lifestyle and can be divided in three main categories: (i) domains functional in ATP generation (16), (ii) domains of unknown function (13), and (iii) other domains (18) (Additional file 7: Table S3).

Category (i) comprises nitrate respiration (Additional file 7: Table S3), and acetate kinase (IPR000890). Furthermore, two domains that are related to ribonucleotide-triphosphate reductase (IPR012833, IPR012840) have been identified which are crucial for this work, as discussed further below. Category (ii), domains of unknown function, must be excluded from the design as nothing can be said about them with the current knowledge.

For the majority of the remaining domains belonging to category (iii) it is not clear how they would contribute to the desired anaerobic lifestyle. For example, several domains associated with siderophore transport (IPR003538) and pilus assembly (IPR008707, IPR013362, IPR013374, IPR025746) are found. While they have been related to virulence in *P. aeruginosa* [33, 34], which is part of its pathogenic lifestyle, they do not appear to be linked to anaerobicity. Other domains may be beneficial but not essential for anaerobic growth. The identified iron-sulfur clusters (IPR007202, IPR018298) for example were absent in the investigated aerobic species. Typically these clusters are oxygen sensitive, which may explain their absence. The iron sulfur clusters have a regulatory role [35, 36] and are implicated as intracellular sensors and in electron transport. Hence, these clusters do not appear essential for a conversion from an aerobic to an anaerobic lifestyle.

### Design of anaerotolerant *P. putida*

Together, the in silico approaches provide a holistic view on the metabolic processes required to enable designing a *P. putida* strain capable of anaerobic growth by fermentation (Additional file 7: Table S3). The main bottlenecks found include l-aspartate oxidase (NadB), dihydroorotate dehydrogenase (PyrD), protoporphyrinogen oxidase (HemY), ribonucleotide-triphosphate reductase (RNRs), and anaerobic energy generation. We address these below.

**Fig. 3:**
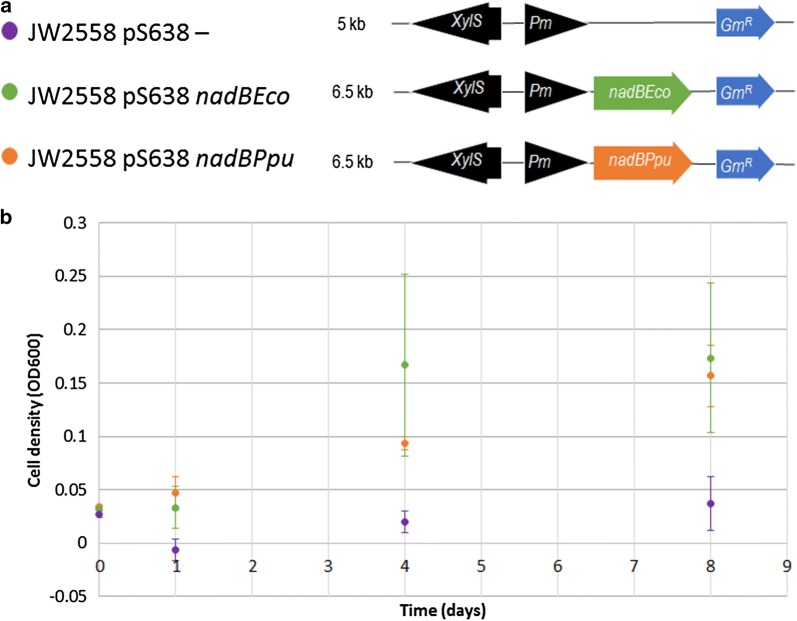
Testing of *P. putida* l-aspartate oxidase under anoxic conditions. **a** Schematic overview of constructs for *E. coli* JW2558: p638—(empty plasmid control) (purple), p638 *nadBEco* (green) and p638 *nadBPpu* (orange). **b** Anaerobic growth analysis of all strains

l*-aspartate oxidase (NadB)* catalyses the conversion of l-aspartate to iminoaspartate, a precursor in NAD(P)^+^ biosynthesis. In iJP962 this conversion requires $\text {O}_2$ as electron acceptor. However, in *E. coli* l-aspartate oxidase is known to use either $\text {O}_2$ or fumarate as electron acceptors [37]. The necessity of exchanging *nadB* in *P. putida* KT2440 must be checked experimentally. This was done by constructing a plasmid with either *nadB* from *P. putida* KT2440 or *nadB* from *E. coli* BW25113 and transforming it into *nadB* knock-out strain* E. coli* JW2558. The growth dynamics of the resulting recombinant *E. coli* strains were compared under anoxic conditions.

*Dihydroorotate dehydrogenase (PyrD)* catalyzes the production of orotate, which is required for pyrimidine biosynthesis, and ultimately for the synthesis of RNA and DNA. In iJP962 this enzyme interacts directly with $\text {O}_2$, but in reality *P. putida* *pyrD* encodes a membrane-bound class II dihydroorotate dehydrogenase [38], which interacts with quinones rather than directly with $\text {O}_2$ [39]. The re-oxidation of the quinones requires the flow of electrons towards the terminal electron acceptor $\text {O}_2$. Thus, *P. putida* *pyrD* is indirectly dependent on $\text {O}_2$ via the electron transfer chain. To circumvent the need for the electron transfer chain, a biochemically characterised class I dihydroorotate dehydrogenase that uses fumarate, FAD^+^, or NAD^+^ [39] could be obtained from the non-pathogenic *L. lactis* [40] and introduced in *P. putida* KT2440.

*Protoporphyrinogen oxidase (HemY)* converts protoporphyrinogen IX to protoporphyrin IX, which is further converted to heme. Heme is involved in many cellular processes—including respiration—and is essential for most organisms, excluding some specific species capable of fermentation under anoxic conditions [41, 42]. It is unclear whether heme is essential for an anaerobically fermenting *P. putida* strain, but it is most likely required for anaerobic respiration. There are two alternative protoporphyrinogen oxidases in gram negative bacteria: HemG and HemJ. The protein HemG is quinone-dependent instead of $\text {O}_2$-dependent, but is not found in any *Pseudomonas* species. The protein HemJ is found in *Pseudomonads*, including *P. aeruginosa* [43] and *P. putida*, but it is unknown whether or not it relies on $\text {O}_2$ [41]. We have thus not included an alternative biosynthesis gene for the heme precursor protoporphyrin IX, as it is likely that protoporphyrin IX can be synthesized by the endogenous *P. putida* HemJ.

*Ribonucleotide-triphosphate reductases (RNRs)* are required for the biosynthesis of deoxynucleotides (dNTPs). There are three classes of RNRs: Class I is strictly aerobic, class II is $\text {O}_2$-independent, and class III is $\text {O}_2$ sensitive [44, 45]. *P. putida* only has a class I RNRs and is not able to produce DNA under anoxic conditions. In contrast, *P. aeruginosa* contains RNR of all three classes [45]. RNR knockout experiments in *P. aeruginosa* revealed that the class II enzyme contributes most to anaerobic dNTP production [45], and that the class II RNRs are reported to be essential for anaerobic growth in *P. aeruginosa* [46]. Therefore, a class II RNR seems the most promising candidate to enable anaerobic nucleotide production in *P. putida*.

The identified limitations and alternative options found using the in silico methods were combined into a consolidated design of a *P. putida* KT2440 strain capable of in-silico growth under anoxic conditions through anaerobic fermentation. The predicted lack of ATP was resolved by acetate production, based on the previously successfully expressed acetate kinase for fermentation [6, 15]. The newly proposed design includes (i) a quinone-independent gene cluster class I dihydroorotate dehydrogenase (*pyrK-pyrD B*) which was taken from the non-pathogenic *L. lactis* [47], (ii) the class III ribonucleotide-triphosphate reductase (*nrdD-nrdG*) which was taken from the non-pathogenic *L. lactis*, (iii) acetate kinase (*ackA*) which was taken from *E. coli*, and possibly (iv) l-aspartate oxidase (*nadB*) from *E. coli* (Fig. 2).

### The *P. putida*l-aspartate oxidase can function without oxygen as electron acceptor

To determine the need to replace the *nadB* gene to facilitate anaerobic survival of *P. putida*, plasmids carrying either *nadB* from *E. coli* (p638 *nadBEco*) or the *nadB* gene from *P. putida* (p638 *nadBPpu*) were inserted into the *E. coli* *nadB* knockout strain *JW2558*. The strains were cultured anaerobically, monitoring growth through sampling. Both strains grow similarly under anoxic conditions, proving that the endogenous l-aspartate oxidase of *P. putida* does not need oxygen as an electron acceptor and therefore does not have to be replaced (Fig. 3).

### Expression of acetate kinase, dihydroorotate dehydrogenase and ribonucleotide triphosphate reductase in *P. putida* does not alter aerobic growth

To verify the in silico analysis, recombinant strains were constructed carrying *ackA*, *pyrK-pyrD B*, *nrdD-nrdG*, or all three (*ackA-(pyrK-pyrD B)-(nrdD-nrdG)*) (Fig. 4a). The genes of interest were placed in the same SEVA pS2213 backbone, with an RK2 origin of replication and an *EcFbFP* gene included directly behind the gene of interest. In the empty plasmid, only the fluorescence gene is present.

A 64-h growth experiment under oxic conditions in a platereader was performed, measuring the $\text {OD}_{600}$ and fluorescence (Fig. 4b–d). The similar growth curves of the different recombinant strains show no difference in plasmid burden, while plasmid sizes vary between 4 kb (empty plasmid) and 10 kb (pS2213 *ackA-(pyrK-pyrD B)-(nrdD-nrdG)*) (Fig. 4b).

All recombinant strains carrying plasmids emit significantly more fluorescence than the wild-type counterpart (Fig. 4c, d). There are large differences in fluorescence between those strains. In 12 h, the relative fluorescence of the strain carrying the empty plasmid was 2.3 to 1.4 times as high as the recombinant strains. At that point, the fluorescence intensity of strain KT2440 pS2213*nrdD-nrdG* exceeds all other strains by a factor 1.6 (Fig. 4d). Remarkably, strain KT2440 pS2213 *ackA-(pyrK-pyrD B)-(nrdD-nrdG)* and strain KT2440 pS2213 *pyrK-pyrD B* emitted similar amounts of fluorescence. In spite of the size of the two-subunit gene cluster class III ribonucleotide triphosphate reductase, relatively more fluorescence was recorded in *P. putida* KT2440 pS2213 *nrdD-nrdG* than when solely acetate kinase is expressed. In this context, the differences in fluorescence between strains can be used as a rough indication of difference in gene transcription, as the genes were placed in the same backbone under de same promoter and with equal copy numbers.

These data show that under oxic conditions all plasmids pose an equal burden, but the level of gene transcription varies significantly between strains.

### Additional ATP generation results in growth of *P. putida* KT2440 under micro-oxic conditions

The recombinant strains were exposed to micro-oxic conditions in an adaptive laboratory evolution set-up using an oxygen gradient first for 2 days, followed by two 4-day cycles. Strain performance was continually monitored using a time-lapse camera, taking a picture every 20 s. The end of one round was marked by either stagnation of growth and visible cell-death, or the ability of the strains to grow under micro-oxic conditions, defined as below the micro-electrode detection limit. Recombinant strains were passed three times over oxygen gradients to adapt the strains to micro-oxic conditions (Fig. 5a). Since KT2440 pS2213 *pyrK-pyrD B* underperformed in preliminary oxygen gradient experiments, this strain was excluded (data not shown). KT2440 pS2213− (negative control), KT2440 pS2213 *ackA*, KT2440 pS2213 *nrdD-nrdG* and KT2440 pS2213 *ackA-(pyrK-pyrD B)-(nrdD-nrdG)* were monitored for three consecutive rounds of culturing in the oxygen gradients. Medium was used as a blank control.

Over multiple consecutive rounds, all recombinant strains except the empty plasmid control showed improved performance under micro-oxic conditions (Fig. 5b). This is reflected by the difference in progress towards a dissolved oxygen concentration of $<0.01$ mg/l along the consecutive passages over the oxygen gradients. In contrast to the other transformant strains, no growth beyond detectable oxygen levels was achieved with the empty plasmid between passage 2 and 3. To ensure that there was no growth possible beyond the detection limit, we deliberately tested the empty plasmid in further cycles. The results show that the empty plasmid control only adapts to the limit of detectable oxygen levels over multiple passages, but does not surpass it (Additional file 8: Figure S2).

After a period of 4 days the strains progressed to areas with $<0.01$ mg/l oxygen (Fig. 5, Additional file 9: Analysis S2, Additional file 11: Analysis S4, Additional file 12: Analysis S5, Additional file 13: Analysis S6, Additional file 14: Document S1 and Additional file 15). While in the set-up, time-wise, the first passage (T1, 2 days) is different from the following cycles (T2 and T3, 4 days), presently it is unclear what causes the sudden increase in performance from T2 to T3 observed in strains carrying either pS2213−, pS2213 *nrdD-nrdG* or pS2213 *ackA*-(*pyrK-pyrD B*)-(*nrdD-nrdG*) (Fig. 5B). When solely acetate kinase is expressed, strain performance in micro-oxic conditions is directly improved. The differences between the strains with pS2213 *ackA* and pS2213 *ackA-(pyrK-pyrD B)-(nrdD-nrdG)* could either be explained by the additional metabolic load caused by gene expression under micro-oxic conditions (which in itself imposes a burden), or by the effect of acetate kinase held back by the additional expression of *pyrK-pyrD B* and *nrdD-nrdG*. When analysing the overall daily progress of the strains (Fig. 5c) it again becomes apparent that overall the strain expressing acetate kinase performs significantly better. Extra energy generation is therewith proven to be a vital requisite for *P. putida* to accommodate micro-aerobic growth.

The results of the $\text {O}_2$-gradients show that after multiple rounds over the oxygen gradient expression of *nrdD-nrdG* alone in *P. putida* KT2440 result in growth under micro-oxic conditions (Fig. 5).

## Discussion

We discuss below the work described along four major lines:

1. The integrated computational analysis herein described provided a coherent, systems-wide basis to understand the factors underlying the aerobic nature of *P. putida*. By enabling to explain previous unsuccessful experimental efforts aiming at making *P. putida* grow under anoxic conditions, this endeavour emphasised the value of applying an *a priori* systems, model-driven design perspective to metabolic engineering, as opposed to the common practice of designing experiments based on fractional knowledge of parts of the system. Also, the combination of GSM simulations and comparative genomics can be of widespread use as they are inherently complementary. GSMs, already available for many organisms [7, 8], describe an organism in high detail; whereas comparative genomics pinpoints the genetic basis underlying the differences between organisms.

**Fig. 4:**
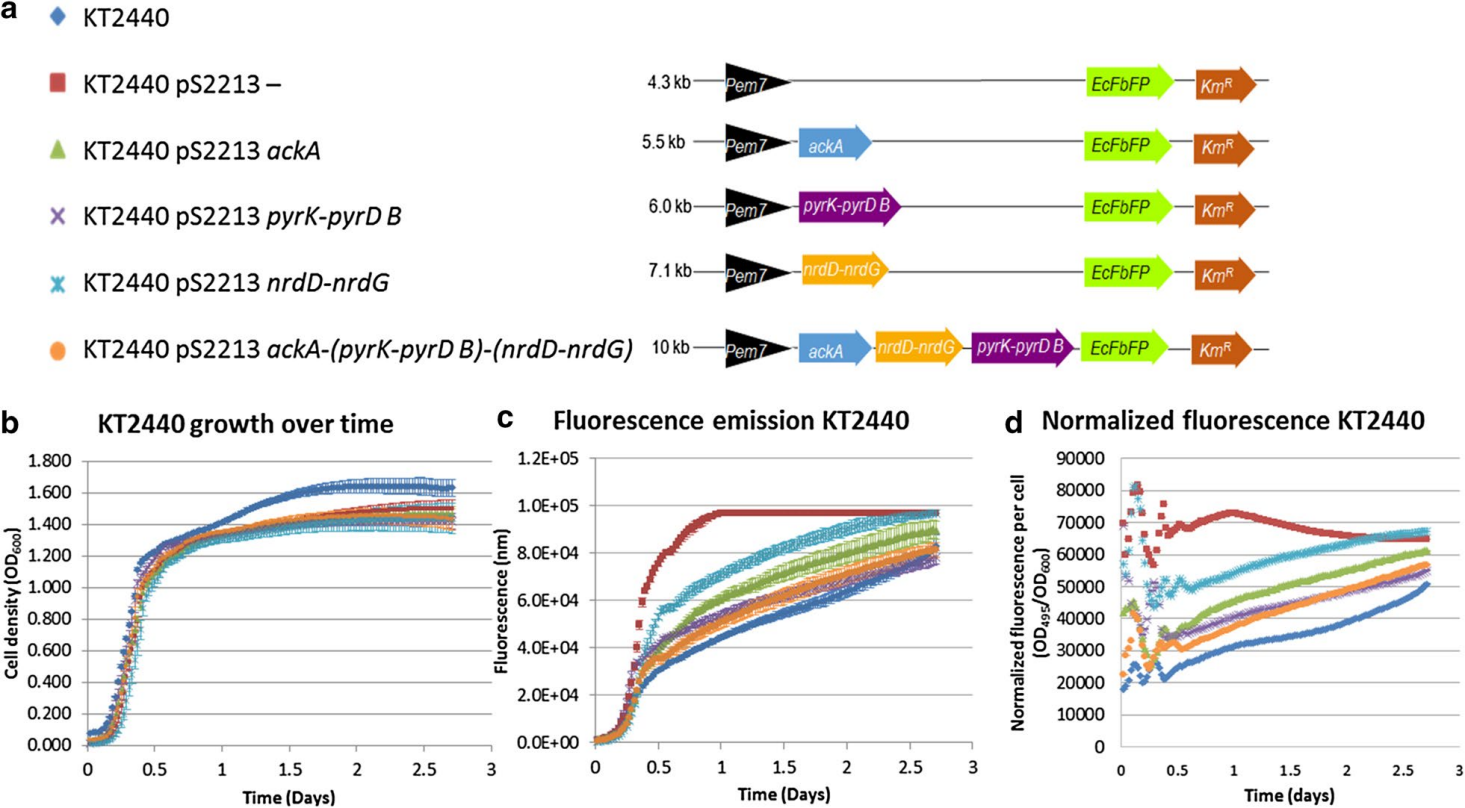
Building the design of *P. putida* KT2440 capable of growth under anoxic conditions and testing under oxic conditions. **a** Schematic overview of constructs for *P. putida* KT2440: 2213F− (empty plasmid control), pS2213 *ackA*, pS2213 *pyrK-pyrD B*, pS2213 *nrdD-nrdG* and pS2213 *ackA-(pyrK-pyrD B)-(nrdD-nrdG)*. The symbols in front of the strain names function as the legend for the graphs. **b** Aerobic growth analysis of all strains. **c** Aerobic fluorescence assay of all strains at excitation of 450 and emission of 495. **d** Fluorescence relative to cell density. Measurements for **b**, **c** and **d** were taken every 30 min for 64 h at $30^\circ \text {C}$. Cultures were shaken continuously
2. According to the understanding of *P. putida* KT2440 metabolism as represented in iJP962, the adjustments we found through comparative genomics and protein domain content analysis should enable anaerobic growth. However, it must be noted that although this metabolic model offers a clear and direct insight into the known metabolism of *P. putida* KT2440, it is far from complete. Due to necessity, metabolic models represent a significantly simplified metabolism. Not enough experimental data is available to describe redox balances and regulatory pathways in a model in full detail, especially considering that much remains unknown about regulatory pathways and protein domain functions. The model contains all reactions known regardless if they are active or not, which means that pathways or reactions that are unlikely to be active under certain conditions are active in the model. The metabolic model therefore offers a good starting point, but should not be considered to provide a perfect design to the ambitious goals we set out. Hence, we rather see the model predictions regarding anaerobic growth as a guide towards achieving micro-oxic robustness.
3. When testing the fermentative design of *P. putida* KT2440 as detailed in Fig. 5, we used adaptive laboratory evolution methods combined with insertion of the genes/gene clusters that enabled the strain to grow under micro-oxic conditions. The oxygen gradient analysis proved a robust and reliable way to follow the progress of recombinant strains versus control strains under micro-oxic conditions within 2 to 4 days’ time over multiple passages. Under oxic conditions, fluorescence levels (Fig. 4) indicate that acetate kinase concentrations in *P. putida* KT2440 pS2213 *ackA* are higher than in KT2440 pS2213 *ackA-(pyrK-pyrD B)-(nrdD-nrdG)*. Under micro-oxic conditions KT2440 pS2213 *ackA* outperforms all other strains, including KT2440 pS2213 *ackA-(pyrK-pyrD B)-(nrdD-nrdG)*. This suggests that acetate kinase directly leads to improved performance under micro-oxic conditions: the more acetate kinase, the larger the ATP availability, the better the strain performance under micro-oxic conditions. However, the accumulation of acetate due to AckA activity could lower the pH to a growth-inhibiting environment. The production of acetate (yielding ATP) could also reduce carbon flow towards the product of interest, lowering the yield. Yet, acetate is just one way to increase the ATP pool. Other options include growing cells on more energy-ready carbon sources such as gluconic acid, knocking out energy demanding pathways [12], applying a different terminal electron acceptor by offering an anode in combination with phenazines or electron mediators in a bioelectrochemical system [17, 18] or switching to anaerobic respiration [16]. The use of genetic switches at the level of acetyl-CoA could also be used to alleviate the potential toxicity exerted by acetate [48]. The experimental set-up with overall improved strain performance in micro-oxic conditions over multiple oxygen gradient passages shows the value of an adaptation set-up in relation to GSM predictions. GSM predictions regarding growth rates and fluxes typically relate better to strains already having undergone adaptive laboratory evolution [49, 50]. Hence, improvement of strain performances under micro-oxic conditions is expected upon further adaptive laboratory evolution.
4. After multiple round over the oxygen gradients, dihydroorotate dehydrogenase and ribonucleotide triphosphate reductase had an added benefit in this assay (Additional files 10, 11, 12, 13, 14 and 15 for raw data and analyses). The ability of growing under micro-oxic conditions of KT2440 pS2213 *nrdD-nrdG* and KT2440 pS2213 *ackA-(pyrK-pyrD B)-(nrdD-nrdG)* after three passages over oxygen gradients, as opposed to *P. putida* KT2440 pS2213−, indicate the added value of these genes. However, pyrK-pyrD B and nrdD-nrdG are expected to be only of value under anoxic conditions, since aerobic endogenous versions of these genes are present in the KT2440 genome. We expect that a switch to anoxic conditions will result in the exclusive use of the anaerobic versions of the proteins.

**Fig. 5:**
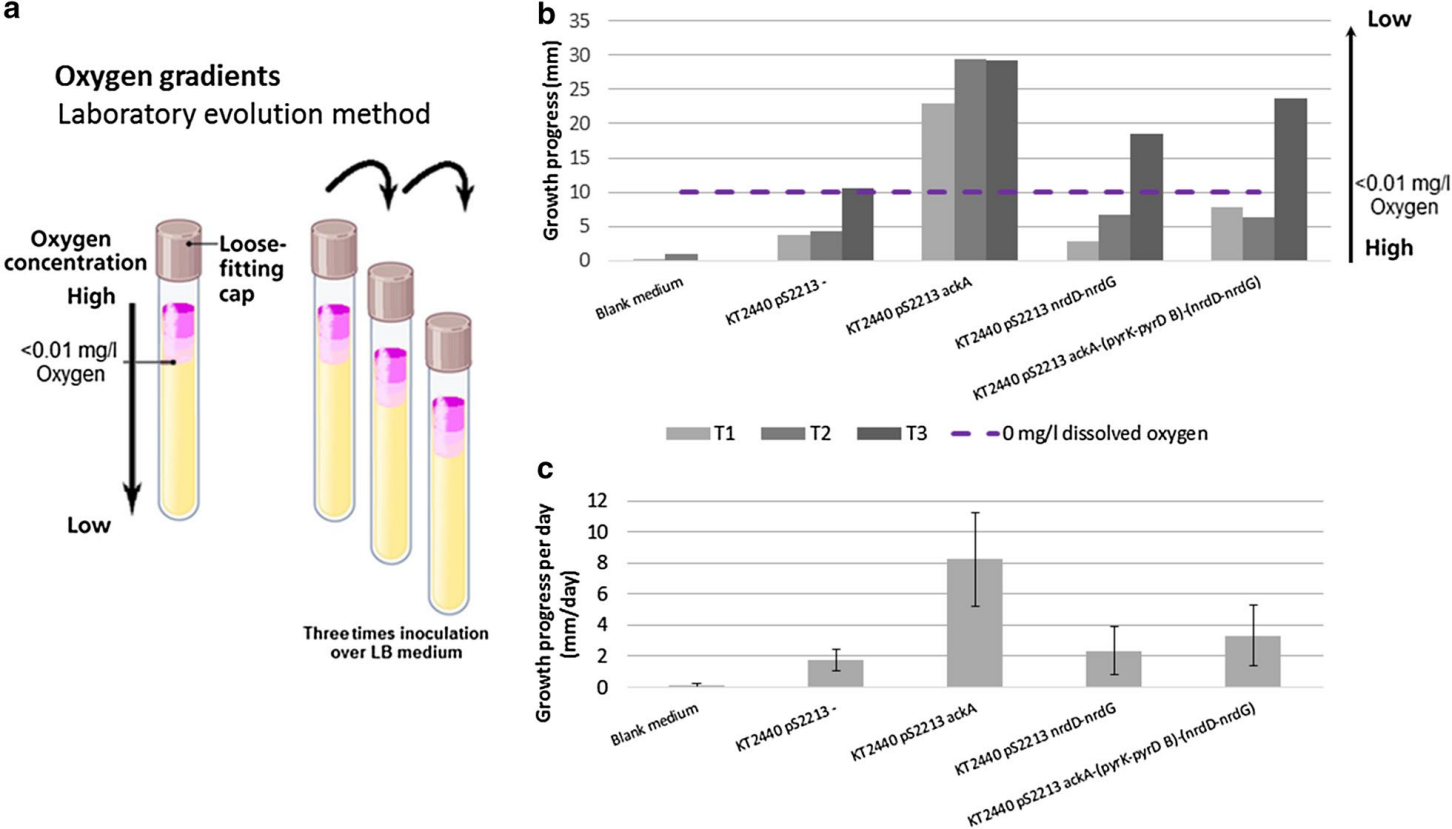
Testing of transformed strains under micro-oxic conditions. **a** Screening assay using an adaptive laboratory evolution method with oxygen gradients. Recombinant strains were passed three times over oxygen gradients to adapt the strains to micro-oxic conditions in LB medium. **b** Ingrowth in oxygen gradients over consecutive rounds. The first adaptation round was monitored over 2 days, followed by two 4-day cycles. Strain performance of *P. putida* KT2440 pS2213−, KT2440 pS2213 *ackA*, KT2440 pS2213 *nrdD-nrdG* and KT2440 pS2213 *ackA-(pyrK-pyrD B)-(nrdD-nrdG)* throughout the experiment was monitored continuously with a time-lapse camera set-up, and was depicted as ingrowth in mm from the surface of the growth medium down. Above the purple dashed line the oxygen concentration is $<0.01$ mg/l. Non-inoculated medium was taken as control. **c** The average progression of the recombinant strains in the oxygen gradients over time, depicted in mm/day

These four items represent as well the core elements of the Design-Build-Test-Learn (DBTL) engineering cycle of Synthetic Biology [51]. This integrated, highly iterative workflow illustrate how such strategies can efficiently assist lifestyle engineering of industrial traits and provide the means to enable a new era in tailored, agile biomanufacturing.

## Conclusions

The obligate aerobic metabolism of *P. putida* is a major obstacle for the breakthrough of this bacterium to be widely used as a biotechnological host. The integrated computational analysis herein described provided a coherent, solid basis to understand the factors underlying the aerobic nature of *P. putida*. The model-driven redesign of an obligate aerobe into a strain tolerant to low-oxygen concentrations constitutes an important fundamental step in the rational engineering of such biological systems.

The initial design addressed five metabolic bottlenecks: protoporphyrinogen oxidase (*hemY*), l-aspartate oxidase (*nadB*), dihydroorotate dehydrogenase (*pyrD*), ribonucleotide-triphosphate reductase (*RNRs*) and anaerobic energy conservation (through substrate-level phosphorylation by acetate kinase, *ackA*). This design could be brought back to the addition of five genes upon further evaluation. Introduction of *hemY* was determined not to be essential as the endogenous HemJ can possibly fulfil its role and *P. putida* NadB was experimentally proven to function under anoxic conditions.

Upon testing the final design under micro-oxic conditions, the increased availability of ATP through the synthesis of acetate in *P. putida* KT2440 pS2213 *acka* results in growth, unlike the wild-type strain. The importance of ATP availability under anoxic conditions was demonstrated earlier [6, 15]. Moreover, an increased ATP pool may also have beneficial effects on other processes, including thermo-tolerance [12, 52]. This work thus confirms that insufficient ATP generation is a main bottleneck for cell growth under oxygen-limiting conditions.

The final design included three enzymes. The two newly introduced gene sets, *pyrK-pyrD B* encoding for class I dihydroorotate dehydrogenase and *nrdD-nrdG* encoding for class III ribonucleotide triphosphate reductase, recognized through the in silico approach, proved effective upon introduction after multiple rounds of adaptive laboratory evolution pressure. This not only highlights the value of the in silico approach, but also demonstrates the importance of design testing under micro-oxic conditions to allow for strain adaptation. Together, these enzymes make up the anaerobic version of the pathway for dNTP production, an essential pathway for strain performance under anoxic conditions.

Combined, they achieve the highest impact, although sole introduction of *nrdD-nrdG* also enabled improved micro-oxic performance on its own. This suggests that *nrdD-nrdG* is the main bottleneck of the two metabolic activities. The impact so far does not compare to the implementation of *ackA*, since the aerobic homologs are still present and functional. We believe that these newly recognized genes will prove to be essential under fully anoxic conditions.

Altogether, this work provides insights into the non-linear process of transforming a strict aerobic species into a facultative anaerobic bacterium.

## Supplementary information

**Additional file 1: Table S1.** Bacterial strains and plasmids used in this study.

**Additional file 2: Table S2.** Primers used in this study to make the constructs described.

**Additional file 3: Table S4.** Development of oxygen gradient assay: testing different types of agar/agaroses.

**Additional file 4: Table S5.** Results of bacterial growth experiments in oxygen gradients testing different types of agar/agaroses. Oxygen gradients were prepared with LB medium + 0.05 g/l l-cysteine + 0.5 g/l sodium thioglycollate + 0.01 g/l resazurin + 6 g/l indicated agar/agarose. The gradients were placed overnight at 4 °C before inoculation to allow the agar/agarose to set.

**Additional file 5: Figure S1.** Additional oxygen gradient experiments of *P. putida* KT2440 pS2213—ingrowth in oxygen gradients over two additional consecutive rounds. The first adaptation round was monitored over 2 days, followed by four 4-day cycles. Strain performance of *P. putida* KT2440 pS2213—throughout the experiment was monitored continuously with a time-lapse camera set-up, and was depicted as ingrowth in mm from the surface of the growth medium down. Above the purple dashed line the oxygen concentration is < 0.01 mg/l.

**Additional file 6: Analysis S1.** Anaerobic cultivation analysis.

**Additional file 7: Table S3.** Overview of identified anaerobic-only protein domains [25]. Indicated are which domains resulting from this in silico analysis were previously introduced in *P. putida* in other studies.

**Additional file 8: Figure S2.** Analysis of resazurin specificity. Multiple oxygen gradients were prepared using M9 minimal medium (A) or LB (B), with agarose concentrations of 1, 2, 3, 4, 5, or 6 g/l. The concentration of dissolved oxygen was determined in mg/l using a micro-electrode. The micro-electrode was moved from high to low oxygen levels by 1 mm/min to ensure stabilization of both the readings and the gradient. The readings were compared to the colour of the resazurin to determine its specificity. The dashed purple line indicates the resazurin colour turning point from pink to colourless.

**Additional file 9: Analysis S2.** Micro-electrode in oxygen gradient analysis.

**Additional file 10: Analysis S3.** Growth and fluorescence analysis.

**Additional file 11: Analysis S4.** Method used for Fiji oxygen gradient analysis.

**Additional file 12: Analysis S5.** Oxygen gradient analysis.

**Additional file 13: Analysis S6.** Additional oxygen gradient analysis.

**Additional file 14: Document S1.** Order of samples from time lapse photos per passage.

**Additional file 15.** Example time lapse movie of oxygen gradients. Time-lapse photos were analysed using FIJI (imageJ64).The complete series are available in Additional file 12: Analysis S5.
