# Supplementary figures and images for: In silico-guided engineering of Pseudomonas putida towards growth under micro-oxic conditions
Source: Microb Cell Fact. 2019 Oct 22;18:179. doi: 10.1186/s12934-019-1227-5 (PMC6805499; doi:10.1186/s12934-019-1227-5)

## Slide 1
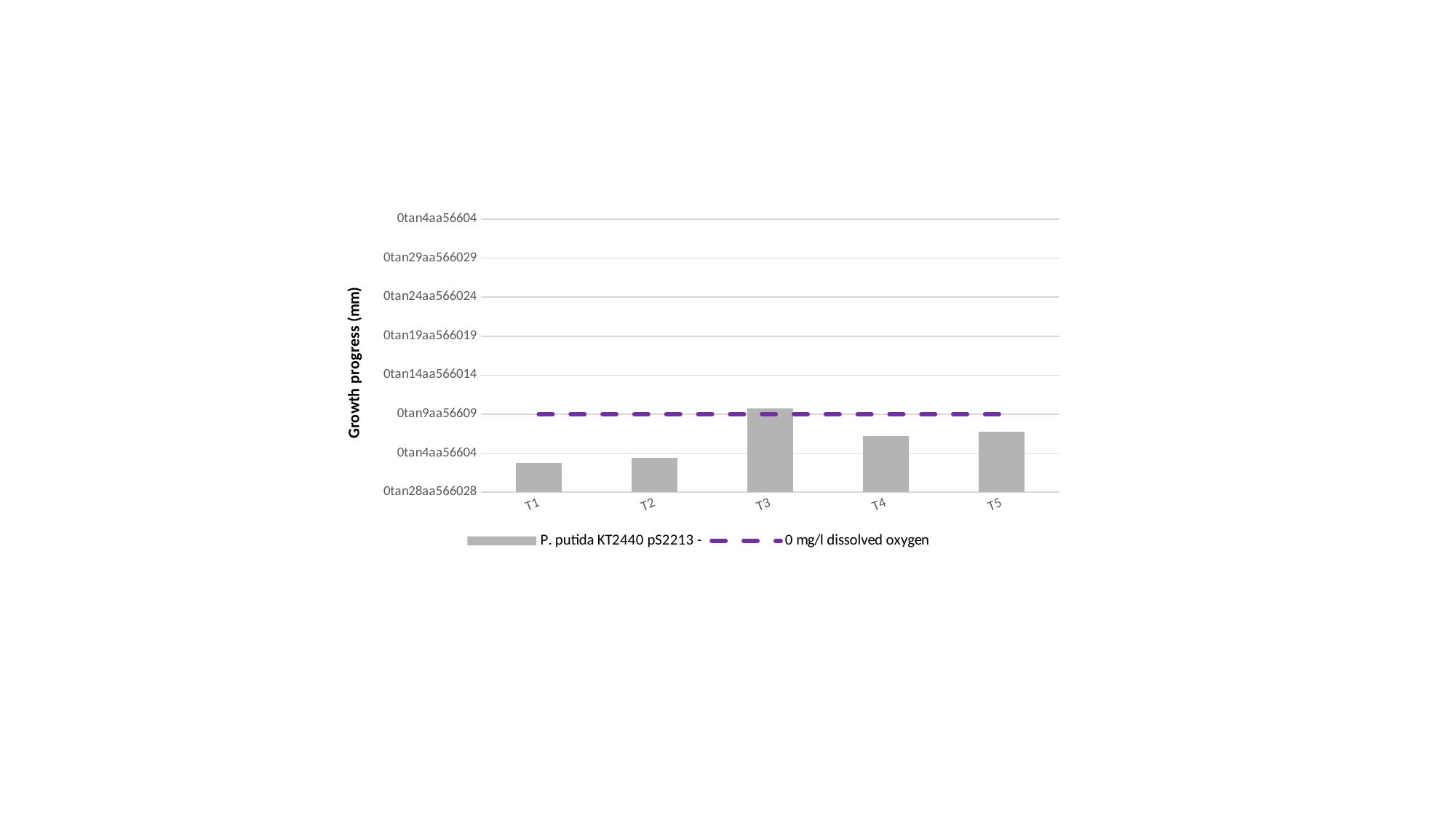

### Chart
| Category | | 0 mg/l dissolved oxygen |
|---|---|---|
| T1 | 3.7142857142857135 | 10.0 |
| T2 | 4.365 | 10.0 |
| T3 | 10.7640873015873 | 10.0 |
| T4 | 7.212837837837838 | 10.0 |
| T5 | 7.736486486486487 | 10.0 |

Supplement: Supplementary file 5 — Additional file 5: Figure S1. Additional oxygen gradient experiments of P. putida KT2440 pS2213—ingrowth in oxygen gradients over two additional consecutive rounds. The first adaptation round was monitored over 2 days, followed by four 4-day cycles. Strain performance of P. putida KT2440 pS2213—throughout the experiment was monitored continuously with a time-lapse camera set-up, and was depicted as ingrowth in mm from the surface of the growth medium down. Above the purple dashed line the oxygen concentration is < 0.01 mg/l. [file 12934_2019_1227_MOESM5_ESM.pptx]

## Slide 1
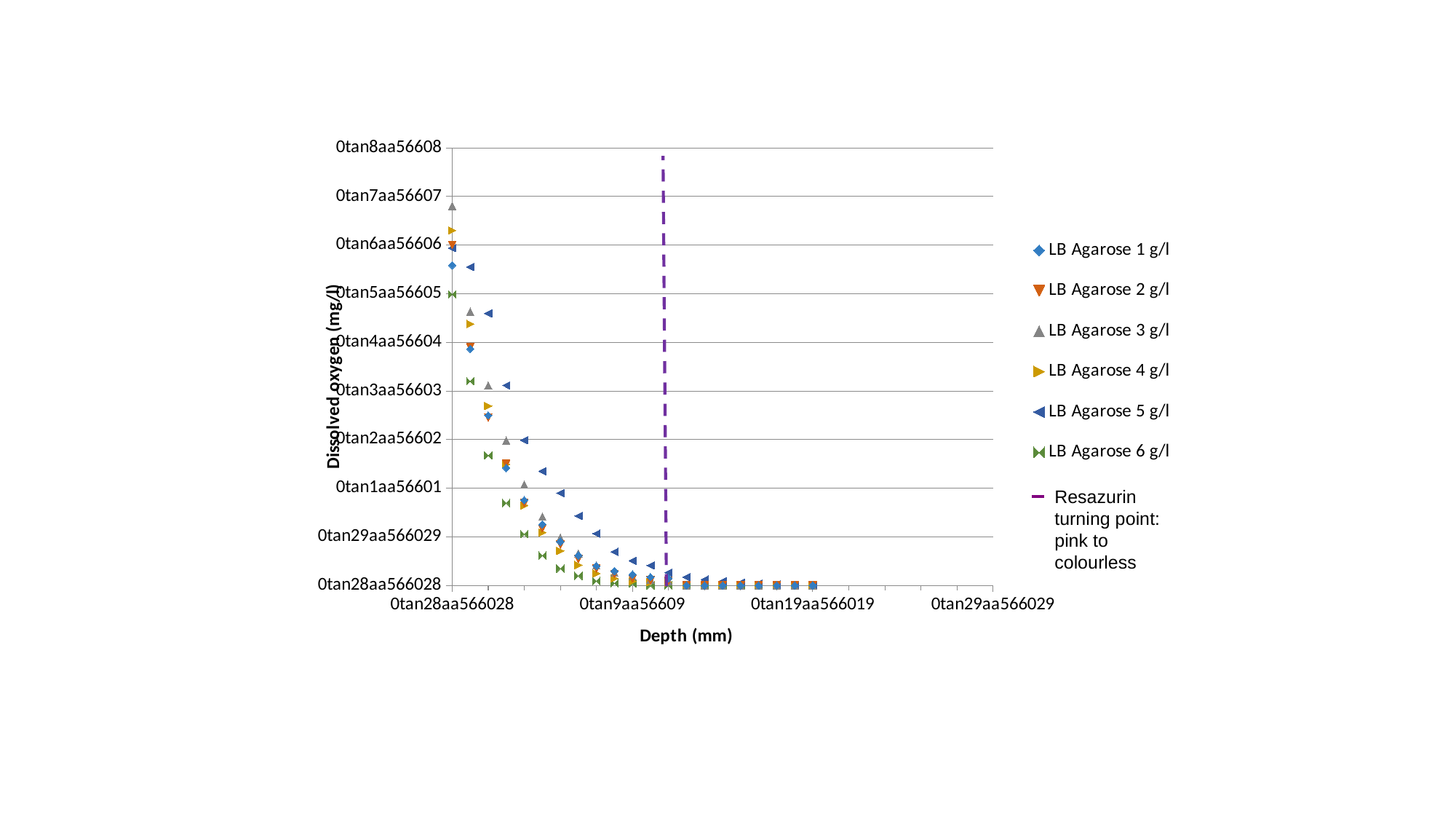

### Chart
| Category | | | | | | |
|---|---|---|---|---|---|---|Resazurin turning point: pink to colourless

Supplement: Supplementary file 8 — Additional file 8: Figure S2. Analysis of resazurin specificity. Multiple oxygen gradients were prepared using M9 minimal medium (A) or LB (B), with agarose concentrations of 1, 2, 3, 4, 5, or 6 g/l. The concentration of dissolved oxygen was determined in mg/l using a micro-electrode. The micro-electrode was moved from high to low oxygen levels by 1 mm/min to ensure stabilization of both the readings and the gradient. The readings were compared to the colour of the resazurin to determine its specificity. The dashed purple line indicates the resazurin colour turning point from pink to colourless. [file 12934_2019_1227_MOESM8_ESM.pptx]

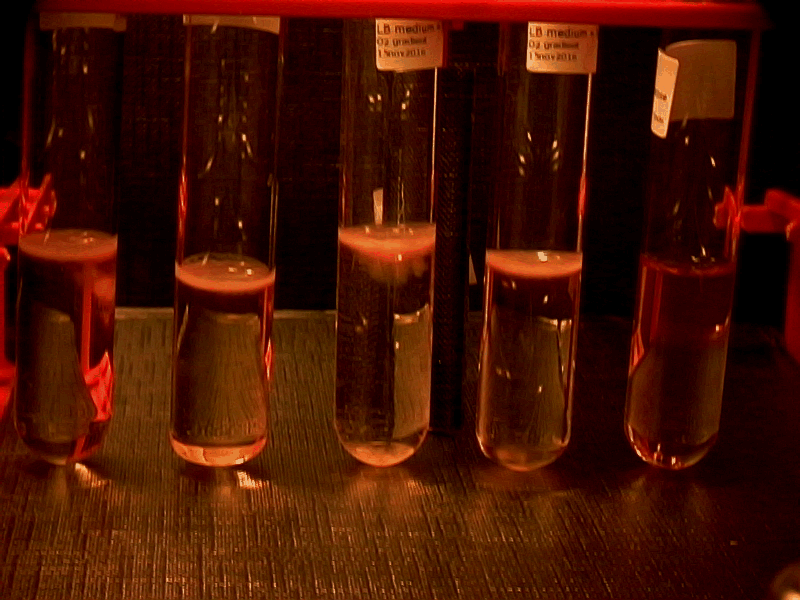

Supplement: Supplementary file 15 — Additional file 15. Example time lapse movie of oxygen gradients. Time-lapse photos were analysed using FIJI (imageJ64).The complete series are available in Additional file 12: Analysis S5. [file 12934_2019_1227_MOESM15_ESM.gif]
