# Supplementary material for: In silico-guided engineering of Pseudomonas putida towards growth under micro-oxic conditions
Source: Microb Cell Fact. 2019 Oct 22;18:179. doi: 10.1186/s12934-019-1227-5 (PMC6805499; doi:10.1186/s12934-019-1227-5)
Supplement: Supplementary file 11 — Additional file 11: Analysis S4. Method used for Fiji oxygen gradient analysis. [file 12934_2019_1227_MOESM11_ESM.docx]

Fiji method for analysis

Time-lapse photos analysed using FIJI (imageJ 64)

> Open virtual imageJ stack

> Image

> type

> 8-bit

> image

> adjust

> threshold [40]_[255]

> black background

> edit

> selection

> specify (each vial specified)

>[20-450-vial specific-260]

> 1 image/20 min

> 210 pixels = 5 cm

> duplicate

> stk

> plot z-axis profile

data analysed in excel
