# Supplementary material for: In silico-guided engineering of Pseudomonas putida towards growth under micro-oxic conditions
Source: Microb Cell Fact. 2019 Oct 22;18:179. doi: 10.1186/s12934-019-1227-5 (PMC6805499; doi:10.1186/s12934-019-1227-5)
Supplement: Supplementary file 14 — Additional file 14: Document S1. Order of samples from time lapse photos per passage. [file 12934_2019_1227_MOESM14_ESM.docx]

Order of analysed strain performance in oxygen gradients left to right:

T1:

1. KT2440 pS2213 *ackA-(pyrK-pyrD B)-(nrdD-nrdG)*
2. KT2440 pS2213 *nrdD-nrdG*
3. KT2440 pS2213 *ackA*
4. KT2440 pS2213 -
5. Blank medium

T2:

1. KT2440 pS2213 *ackA-(pyrK-pyrD B)-(nrdD-nrdG)*
2. KT2440 pS2213 *nrdD-nrdG*
3. KT2440 pS2213 *ackA*
4. KT2440 pS2213 -
5. Blank medium

T3:

1. KT2440 pS2213 –
2. KT2440 pS2213 *nrdD-nrdG*
3. KT2440 pS2213 *ackA*
4. KT2440 pS2213 *ackA-(pyrK-pyrD B)-(nrdD-nrdG)*
5. Blank medium
